# Modeling of Sensory Properties of Poppy Sherbet by Turkish Consumers and Changes in Quality Properties during Storage Process

**DOI:** 10.3390/foods12163114

**Published:** 2023-08-18

**Authors:** Behiye İncisu Aydoğdu, Nazan Tokatlı Demirok, Seydi Yıkmış

**Affiliations:** 1 Department of Nutrition and Dietetics, Tekirdağ Namik Kemal University, Tekirdag 59030, Turkey; incisuaydogdu59@gmail.com (B.İ.A.);; 2 Department of Food Technology, Tekirdag Namık Kemal University, Tekirdag 59830, Turkey

**Keywords:** poppy sherbet, sensory properties, bioactive compounds, phenolic compounds, antioxidant capacity, RSM

## Abstract

Poppy is an important edible plant containing bioactive components. This study aimed to produce good-tasting poppy sherbet by determining the content using a response surface methodology (RSM). At the same time, bioactive components, phenolic compounds, and color properties were investigated in optimum poppy sherbet during storage; 0.26 g of dried corn poppy flowers, 0.15 g of citric acid, and 4.29 g of sucrose values were the most promising, achieving high scores for color, smell, taste, and general acceptance from sensory properties (sensory score of 8.55 for color; 7.19 for smell; 8.38 for taste; 7.98 for general acceptability). A total of nine polyphenols were detected in the optimum poppy sherbet sample; gallic acid was the most common. There was no statistically significant difference between the samples stored on the 0th and 30th days regarding gallic acid content (23.886 ± 0.164 μg/mL, 23.403 ± 0.343 μg/mL) and protocatechuic acid (1.146 ± 0.048 μg/mL, 1.047 ± 0.038 μg/mL). Total flavonoid contents (TFC), total phenolic contents (TPC), CUPRAC (cupric ion reducing antioxidant capacity), DPPH (e free radical diphenylpicrylhydrazyl), total monomeric anthocyanin (TAC), and color values were found to decrease as the storage period increased. It was considered that a highly palatable and rich bioactive component product could be obtained.

## 1. Introduction

Due to the harmful consequences of synthetic substances, consumers have chosen to consume food products containing natural ingredients. Edible flowers have traditionally been defined as non-toxic flowers that may be consumed in the human diet; they help to produce food for the body and have other health advantages [1]. Wild plants, which are rich in minerals, vitamins, and proteins and have high antioxidant capacities, are preferred by people living in rural areas in Turkey [2]. It can also be a good alternative to plant extracts, preservatives, and chemical pesticides [3].

The family *Papaveraceae* includes 44 genera and approximately 825 herbaceous plant species and is distributed in the temperate regions of the Northern Hemisphere, in the western parts of South America and Southern Africa [4]. Poppy (*Papaver rhoeas* L.), one of the plants that is the cornerstone of the ecosystem and is widespread worldwide, is a member of the *Papaveraceae* family [5]. It is thought that the name of the poppy was given because of the red color of the wedding dress in the old Turkish customs and the color of the flowers of the poppy plant, which is similar to the wedding dress and its elegance [6]. It is reported that the poppy grows most commonly on limestone soils and is found in the wild in Europe [7]. The poppy is reported to be developed in Western Asia, Pakistan, and North Africa (outside of Europe) [8]. Poppy flowers are used as a source for tea and food coloring [9]. The stem, seeds, and petals of the poppy can be consumed as food, while the stem parts of the poppy are used in salads and the petals are used in making poppy sherbet [2]. The poppy has been noted to contain the flavonoids quercetin, kaempferol, luteolin, and hypolaetin and their glycosides 3-O-b-D-glucopyranosyl kaempferol (astragalin), 3-O-b-D-glucopyranosyl quercetin (isoquercetin), and 3-O-beta-D-galactopyranosyl quercetin (hyperosyl) [10], several flavonols such as kaempferol, isorhamnetin, quercetin, and myricetin, as well as minerals such as calcium, potassium, and sodium [8]. The poppy, which is very common in Anatolia, also contains sugar, mucilage, and gum in its petals [11,12]. Dogan et al. (2014) determined that sesquiterpene hydrocarbons were the main group in *Papaver rhoeas*. They found essential oil composed of phytol (52.8%), tricosane (7.8%), 2-pentadecanone (6%), and heneicosane (5.3%) from the plant, respectively [13]. Renna et al. (2015) indicated that *P. rhoeas* could be considered a good source of Fe and Mn [14]. Additionally, recent studies have demonstrated that poppies and their extracts have antibacterial, antimutagenic, anti-inflammatory, antiulcerogenic, antidepressant, cytoprotective, anti-analgesic, and antioxidant activities [15]. A study that investigated antimicrobial activities of twelve different alkaloids belonging to promorphinan (salutaridine), aporphine (roemerine), rhoeadine (epiglaucamine, glaucamine, glaudine, isorhoeadine, isorhoeagenine, rhoeadine, and rhoeagenine), protopine (coulteropine and protopine), and proaporphine (mecambrine) groups were isolated from *Papaver rhoeas* L., found that aporphine (roemerine) showed significant antimicrobial activity against *Candida albicans* with a MIC value of 2.4 µg/mL and against *Staphylococcus aureus* with a MIC value of 1.22 µg/mL [9]. In another study, sinactine, stylopine, epiberberine, canadine, and berberine isolated from Lebanese *Papaver rhoeas* showed dose-dependent inhibitory effects, with highest activity for human colon cancer cells (HCT116), breast cancer cells (MCF7), the human keratinocyte cell line (HaCaT), and non-cancerous colon cells (NCM460) [16]. Marsoul et al. (2020) reported that soxhlet extract from *Papavera rhoeas* showed antibacterial activity against *Enterobacter feacalis*, with a MIC value of 0.11 mg/mL [17].

In the literature review, there are a few studies regarding safety factors of the poppy flower [18]. Günaydın et al. (2015) stated in five case reports that patients had myosis, seizures, CNS depression, and confusion due to unconscious overconsumption of *Papaver rhoeas* L. [19]. Because of possible intoxication by alkaloids, it is suggested to eat leaves in moderation. Also, high concentrations (25 mg/mL) from the flowers of corn poppy gave potent genotoxic and cytotoxic effects [18]. Cadmium causes adverse health effects in humans and is considered one of the most toxic heavy metals. Poppy inreases the risk of cadmium accumulation [20]. In the studies, Herbicide Resistance Action Committee B (active ingredients: chlorosulfuron, florasulam, iodosulfuron-methyl-Na, metsulfuron-methyl, rimsulfuron, tribenuron-methyl, and thifensulfuron-methyl) and Herbicide Resistance Action Committee O (active ingredients: aminopyralid, clopyralid, dicamba, MCPA, MCPB, and mecoprop) groups have been reported to dominate the EU market with *Papaver rhoeas* L. [21]. Due to *Papaver rhoeas* L.’s herbicide-resistant biotype, many articles have been published on this topic in recent years [21,22,23,24,25].

Response surface methodology (RSM) uses mathematical and statistical methods to optimize experimental parameters, cut down on the number of treatments, and identify the impacts of experimental parameters [26]. As opposed to conventional experimental approaches, RSM provides a greater understanding of the process, making it the most common method for multivariate statistical techniques. It evaluates intricate processes involving the interaction of variables that might determine how inputs affect outcomes [27].

The purpose of the present study was to improve the taste of poppy sherbet and to determine the ingredient by the RSM method, which has not been found in the literature before. In addition, we evaluated the bioactive compounds, phenolic content, antioxidant activity, and color properties of optimized poppy sherbet. This study aimed to create a product with high likeability, while also being rich in bioactive components, for consumers who are increasingly looking for healthy and delicious products.

## 2. Materials and Methods

### 2.1. Preparation and Thermal Pasteurization of Poppy Sherbet

The black parts on the leaves of the poppies collected in the Tekirdağ region were removed with the help of scissors. Abidoye et al. (2022) described a method with some modifications to prepare poppy sherbet. After washing the poppy leaves, they were dried in a hot oven at 40 °C for 12 h and stored. Poppy sherbet was prepared using sucrose, citric acid, and poppy leaves. Poppy sherbet samples were pasteurized for 2 min in a water bath (Wisd-Model, WUC-D06H, Daihan, Republic of Korea) at 85 ± 1 °C [28].

### 2.2. Modeling Procedure for Response Surface Method

The surface response method Minitab Statistical Analysis Software (Minitab 18.1.1) was used to understand the effect of the preparation of the poppy sherbet recipe on sensory parameters. Central composite design was chosen and a three-level three-factor experimental design was created (Table 1). There were 15 trial points for optimization (Table 2). Model adequacy was evaluated by considering the R^2^ and adjusted-R^2^ coefficients, lack-of-fit tests, and ANOVA results. Independent variables were determined as poppy (X_1_), sucrose (X_2_), and citric acid (X_3_). Color, smell, taste, and general desirability were chosen as dependent variables. The levels of the variables used in the study are shown in Table 1; −1 represents the minimum value, 0 is the center value, and 1 the maximum value. Poppy sherbets according to the experimental design specified in Table 1: minimum (−1) 0.1 g and maximum (1) 0.3 g dried poppy (X_1_), minimum (−1) 3 g and maximum (1) 5 g sucrose (X_2_), and minimum (−1) 0.05 g and maximum (1) 0.15 g citric acid (X_3_). Sensory analysis was carried out after the poppy sherbets were brought to 4 °C for sensory analysis.

The second-degree polynomial (Formula (1)) equation was used to determine the relationship between responses (color, taste, smell, and general acceptance) and independent variables.

(1) $$y=\beta_{0}+\sum_{i=1}^{3}\beta_{i}X_{i}+\sum_{i=1}^{3}\beta_{ii}X_{i}^{2}+\sum_{\begin{array}{c} i=1\\ i<j \end{array}}^{3}\sum_{j=1}^{3}\beta_{ij}X_{i}X_{j}$$

The definition of this formula is as follows: the dependent variable (y); the intercept term (β_o_); the first order (linear) equation coefficient (β_i_); the quadratic equation coefficient (β_ii_); the two-factor cross-interaction coefficient (β_ij_); X_i_ and X_j_ the independent variables.

### 2.3. Determination of Color Parameters

The samples’ color were determined using the liquid container and the Color Meter PCE-CSM 5 colorimeter. Color was specified from the type of color parameters L* (darkness–lightness), a* (greenness–redness), and b* (blue–yellowness). The device was calibrated with a calibration plate before measurements [29]. Chroma (C), h (hue angle), and ΔE (total color change) values were calculated with the following Formulas (2)–(4) [30].

C = (a^2^ + b^2^)^1/2^ (2)

h = tan^−1^(b/a) (3)

ΔE = ((ΔL)^2^ + (Δa)^2^ + (Δb)^2^)^1/2^ (4)

Analyses were carried out in three parallels [31].

### 2.4. Sensory Analysis

Approximately 50 mL of the poppy sherbet samples in amber-colored glass bottles were stored at 4 °C for up to 0, 10, 20, and 30 days of the study. The sensory properties were determined by a method described by Petrou et al. (2012), with some modifications. Sherbets were evaluated by 80 non-trained consumers (62 women, 18 men). Panelists evaluated each sample’s color, smell, taste, and general acceptance [32]. All models were coded using random three-digit numbers [33]. Analyses were carried out between 10.00 and 13.00, which were the most suitable times for sensory testing during daylight. The sensory test environment was physically prepared in such a way that the panelists would not be affected by each other. Before the sensory analysis, the panelists were informed about how to do the pre-tasting and tasting processes. The samples were randomly numbered with 3-digit numbers. There was also random order in the serving of the samples. Sensory analyses were made during daylight in the laboratory environment. After tasting the samples, the panelists were served water to refresh their sense of taste and to rinse their mouths. Sensory characteristics were determined using a 9-point hedonic scale (0–9). Scale scores were as follows: excellent, 9; very good, 8; good, 7; acceptable, 6; poor (first odorless, tasteless development), <6; sub-score 6 was accepted. The product was identified as unacceptable after initial odor or taste [32].

### 2.5. Total Phenolic Contents and Total Flavonoid Contents

The total amount of phenolic (TPC) substances was determined spectrophotometrically by modifying the Folin–Ciocalteu method [34]. The solution was prepared by taking 0.1 mL of poppy sherbet and completing it with 4.5 mL of distilled water. Then, Folin–Ciocalteu reagent (0.1 mL) was added to the sample, vortexed for 1 min to make it homogeneous, and left for 3 min. A saturated sodium carbonate solution (0.3 mL) was added and vortexed. After waiting for 2 h, the absorbances of the samples were read by SP-UV/VIS-300SRB spectrophotometer at 765 nm wavelength. Results were expressed as gallic acid equivalents (GAE). Analyses were carried out in three parallels.

Total flavonoid content (TFC) was determined by the aluminum chloride (AlCl_3_) colorimetric analysis method. Dissolve 1 mL of extracts or 5 mg of quercetin in 1 mL of ethyl alcohol (80%) in a 10 mL volumetric flask containing double-distilled water (4 mL) was added, then diluted (50, 100, 150, 200, 300, 400 mg/L). Then, 5% NaNO_2_ (sodium nitrite) (0.3 mL) was added to the bottle; after 5 min, AlCl_3_ (10%) (0.3 mL) was added. At 6 min, 2 mL of NaOH (sodium hydroxide) (1 M) was added; the total volume was brought to 10 mL with double-distilled water. The solution was thoroughly mixed and measured against space at an absorbance level of 510 nm. Total flavonoid content was expressed as mg catechin equivalents (CE) per liter [35]. Experiments were performed with three replications of total flavonoid analysis and the results were averaged.

### 2.6. Determination of Total Antioxidant Capacity by DPPH

To determine the ability of the samples to reduce the DPPH (2,2-diphenyl-1-picrylhydrazil) free radical, a solution of DPPH prepared in 4.9 mL of ethanol (0.1 M) was added to 0.1 mL of sample or 80% ethanol for control. After waiting for 30 min in a dark condition at room temperature, measurements were made at a wavelength of 517 nm using a UV/VIS spectrophotometer device (SP-UV/VIS-300SRB) [36]. The following Formula (5) calculated DPPH percent inhibition.

DPPH radical scavenging activity (%) = (A_0_ − A_1_)/A_0_) × 100 (5)

where A_0_ is the absorbance of the control and A_1_ is the absorbance of the sherbet.

### 2.7. Determination of Total Antioxidant Capacity by CUPRAC

The total antioxidant activity of the poppy sherbet samples was determined by measuring the ability of antioxidants to reduce copper ion with the CUPRAC method (copper reducing antioxidant capacity). An amount of 100 µL of the prepared poppy sherbet was mixed with 1 mL of 10 mM CuCl_2_.2H_2_O (copper (II) chloride dihydrate) solution, 1 mL of 7.5 mM neocuproine solution, and 1 mL of 1 M ammonium acetate (pH = 7) solution, respectively. After adding 1 mL of water and waiting for 30 min, the absorbance values against the blank were read at 450 nm wavelength. The result was expressed as µmol Trolox g^−1^ [37,38]. Analyses were carried out in three parallels. The following Formula (6) calculated CUPRAC percent inhibition.

CUPRAC (%) = (A_0_ − A_1_)/A_0_) × 100 (6)

where A_0_ is the absorbance of the control and A_1_ is the absorbance of the sherbet.

### 2.8. Determination of Total Monomeric Anthocyanin

Total monomeric anthocyanin (TAC) was determined by the pH differential method. After 1 mL of ferret sherbet samples were taken into two different tubes, pH: 1.0 pH buffer was added to the first tube and 9 mL of pH: 4.5 pH buffer was added to the second tube. The vortexed dilute samples were kept in the dark for 15 min. Afterward, the absorbance values of the samples against water at 510 nm and 700 nm wavelengths were measured using SP-UV/VIS-300SRB spectrophotometer. The absorbance difference value of the diluted sample was calculated with the help of Formulas (7) and (8) [39,40].

Total monomeric anthocyanin (mg/L) = A MW Df 1000/(Ɛ) ℓ(7)

A = (A_λvis_-max − A_700_)pH _1_._0_ − (A_λvis_-max − A_700_)pH _4.5_ (8)

MW = cyanidin-3-glucoside (cyd-3-glc) molecular weight: 449.2 (gmol/L);

Df = dilution factor;

Ɛ = cyanidin-3-glucoside absorption coefficient (26,900 L/(cm mol));

ℓ = lightpath (1 cm).

### 2.9. Phenolic Compounds (HPLC-DAD)

Analysis of phenolic compounds was performed with an Agilent 1260 Infinity chromatograph (C-18, ACE Generix column (250 × 4.6 mm; 5 µm packaging; Agilent) (Advanced Chromatography Technologies Ltd., Aberdeen, Scotland)) equipped with a diode array detector. The column flow rate was adjusted to 0.80 mL/min, and the temperature was fixed at 30 °C. Solution A (water with phosphoric acid (0.1%)) and Solution B (acetonitrile) were used for gradient elution; 17% B (0 min), 15% (7 min), 20% (20 min), 24% (25 min), 30% (28 min), 40% (30 min), 50% (32 min), 70% (36 min), and 17% (40 min) were used as the gradients. The injection volume was prepared at 10 µL to analyze phenolic compound fractions. Phenolic compounds were identified based on UV-Vis data obtained from authentic standards and retention times of the pure compounds available (280, 320, and 360 nm). Concentrations were expressed in µg/mL [41].

### 2.10. Statistical Analysis

Results were presented as means standard deviation (SD), with each experiment being run in triplicate. The study’s statistical analyses were carried out with SPSS 22.0 (SPSS Inc., Chicago, IL, USA). For the analysis of variance, Levene’s Test was applied and a variance homogeneity test was performed. The one-way ANOVA multiple comparison-Tukey tests were used to compare the samples; 0.05 was chosen as the statistically significant level. The OriginPro version 2020 b (OriginLab, Northampton, MA, USA) was used to determine Pearson’s correlation coefficients.

## 3. Results

The results of the regression analysis’s mathematical Formula (9) show the influence of the independent variables on the colors of the sherbet made from poppies.

(9) $$Color=0.14+30.9X_{1}+1.619X_{2}+8.78X_{3}-44.13X_{1}X_{1}-0.1305X_{2}X_{2}-2.6X_{3}X_{3}-1.473X_{1}X_{2}-9.4X_{1}X_{3}-0.70X_{2}X_{3}$$

An increase in the amounts (g) of X_1_, X_2_, and X_3_ positively influenced the color outcomes of the poppy sherbet, according to the formula, showing a linear effect. Additionally, it was noted that the poppy sherbet’s color findings were adversely affected by the squared effects and interaction effects of these independent variables. The effect of the independent factors on the outcomes of the poppy sherbet smell was provided by the mathematical Formula (10), produced from the regression analysis.

(10) $$Smell=-2.063+28.12X_{1}+2.093X_{2}+18.06X_{3}-44.98X_{1}X_{1}-0.1817X_{2}X_{2}-47.0X_{3}X_{3}-1.311X_{1}X_{2}+3.61X_{1}X_{3}-1.523X_{2}X_{3}$$

**Figure 1:**
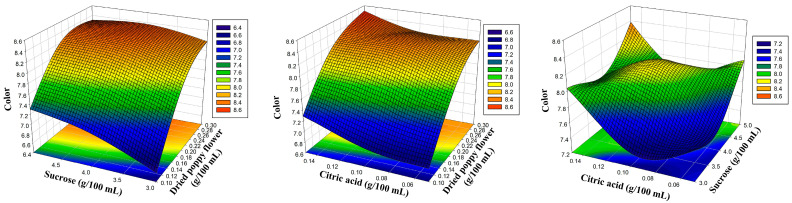
Three-dimensional response surface plots representing the effect of process variables on color.

**Figure 2:**
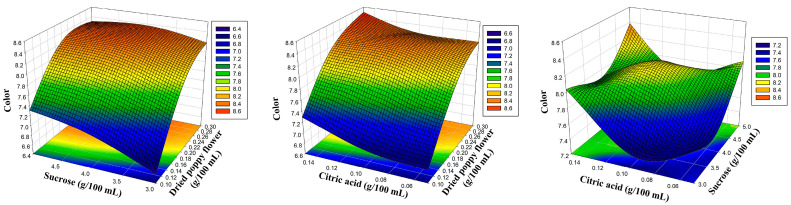
Three-dimensional response surface plots representing the effect of process variables on smell.

**Figure 3:**
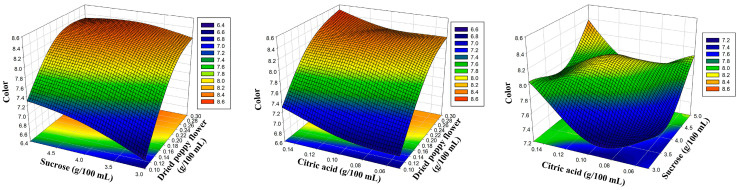
Three-dimensional response surface plots representing the effect of process variables on taste.

**Figure 4:**
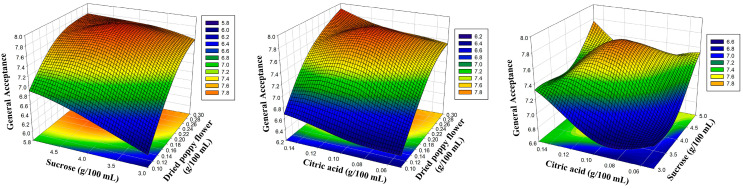
Three-dimensional response surface plots representing the effect of process variables on general acceptance.

The amounts of dried poppy flower, sucrose, and citric acid were chosen as the experiment’s coded independent variables, as indicated in Table 1. To more clearly depict the influence of the sensory characteristics of poppy sherbet, three-dimensional response surface plots were created (Figure 1, Figure 2, Figure 3 and Figure 4). The results were balanced across the four sensory criteria, as seen in the figure. To achieve an optimum poppy sherbet for the dependent variables of color, flavor, aroma, and general acceptance, 15 trial points were undertaken (Table 2).

When the linear impacts of the quantities (g) of X_1_, X_2_, and X_3_ were increased, it was seen that the poppy sherbet’s aroma improved, according to the formula. However, the poppy sherbet’s fragrance results were adversely affected by the squared effects and interaction effects of these independent variables. Contrarily, it was discovered that the binary interaction of citric acid and dried poppy flowers positively affected the fragrance outcomes of the poppy sherbet.

The mathematical Formula (11) obtained from the regression analysis provided the effect of the independent variables on the taste outcomes of the poppy sherbet.

(11) $$Taste=-0.43+29.37X_{1}+1.699X_{2}+15.80X_{3}-44.05X_{1}X_{1}-0.1234X_{2}X_{2}-26.2X_{3}X_{3}-1.508X_{1}X_{2}+4.7X_{1}X_{3}-1.78X_{2}X_{3}$$

The findings of the poppy sherbet’s taste tests were positively impacted by an increase in the linear effects of the quantities (g) of X_1_, X_2_, and X_3_, according to the formula. However, the poppy sherbet’s flavor findings were adversely impacted by the squared effects and interaction effects of these independent variables. However, it was discovered that the poppy sherbet’s flavor was favorably influenced by the binary interaction between citric acid and dried poppy flowers. The results of the regression analysis are presented in mathematical Formula (12), which shows the influence of the independent variables on the general acceptability results of poppy sherbet.

(12) $$\mathrm{General}\;\mathrm{Acceptance}=-2.404+30.24\;X_{1}+2.463X_{2}+18.83X_{3}-47.86X_{1}X_{1}-0.2273X_{2}X_{2}-64.9X_{3}X_{3}-1.433X_{1}X_{2}+4.68X_{1}X_{3}-0.892X_{2}X_{3}$$

A closer look at the formula reveals that an increase in the linear effects of the quantities (g) of X_1_, X_2_, and X_3_ positively influenced the poppy sherbet’s general acceptability outcomes. However, the squared and interaction effects of these independent variables negatively impacted the general acceptance results of the poppy sherbet. Nonetheless, it was concluded that the binary interaction between dried poppy flowers and citric acid positively influenced the general available results of the poppy sherbet. Li et al. (2021) reported that similar cordyceps flower beverages tasted pleasant and rounded with citric acid [42].

The analysis of variance (ANOVA) and regression analysis results are provided for the color, smell, taste, and general acceptance values of the poppy sherbet in Table 2 and Table 3. The color, smell, taste, and general acceptance values of the poppy sherbet were shown to be strongly influenced by the linear effects of the independent variables X_1_, X_2_, and X_3_ (*p* < 0.05). The squared effect of the independent variable X_1_ had a statistically significant impact on the color, smell, taste, and general acceptance levels of the poppy sherbet (*p* < 0.01). The R^2^ values were determined to be 98.23% for color, 99.40% for smell, 98.44% for taste, and 99.75% for general acceptance. The high F-value (221.58) and low *p*-value (<0.0001) for the general acceptance parameter indicated that the model was highly consistent.

Optimum amounts of independent variables were determined to obtain optimized poppy sherbet in Table 4 (the amount of dried poppy flower was 0.26 g, the amount of sucrose was 4.29 g, and the amount of citric acid was 0.15 g). In these conditions, it was determined that the general acceptance result was 7.98, the taste result was 8.38, the smell result was 7.19, and the color result was 8.55 in the sensory analysis. The values of the poppy sherbet obtained as a result of RSM and the repeated experimental values were compared. As a result, the approximate estimation values of color, smell, taste, and general acceptance results were determined as 99.50%, 96.90%, 99.30%, and 98.71%, respectively.

### 3.1. Sensory Properties in the Storage Process

Table 5 compares the sensory analysis ratings for the analyzed optimized poppy sherbet samples at the 0th, 10th, 20th, and 30th days. After a 30-day storage period, it was discovered that the optimized poppy sherbet’s color, smell, and taste results from the sensory study dramatically decreased when compared with the 0th-day sample (*p* < 0.05). Palka and Wilczyńska (2023) determined that the organoleptic properties (overall preference, color, odor, taste, and consistency) of blueberry, strawberry, raspberry and passion fruit–mango sorbets decreased during storage [43] (similar results to those found in our study). Hipólito et al. (2016) reported that, based on consumers’ evaluation, there was little change in the acceptance of lemon, orange, melon, strawberry, and mango sorbets during storage at −18 °C for 21 days [44]. This different result may have been due to the storage of the sorbets at −18 °C. The general acceptance level gradually decreased during the 30-day storage period, but this decrease was not statistically significant (*p* > 0.05). Based on the sensory analysis of the optimized poppy sherbet, it was determined that the highest preference level was observed in the 0th-day sample. In contrast, the lowest likability level was observed in the 30th-day sample. Ekici et al. (2018) found that the favorite sorbet in terms of color among the sorbet samples (rose, unripe grape, evil eye, linaria, engagement, and sirkecunbin) was engagement sorbet (7.33) [45]. In a study conducted by Yıkmış et al. (2020) on sirkencübin syrup, it was observed that the sensory analysis points’ result of the sirkencübin syrup showed a decrease in color after 30 days of storage compared with the 0th day, while the smell and taste results increased [46]. The decrease in color results was observed to be similar between our study and the study mentioned. In a study by Loizzo et al. (2022) on virgin olive oil with apple vinegar, it was found that storage (180 days) did not influence the sensory properties of the products, except for appearance [47]. In a study by Matabura and Kibazohi (2021) on mixed fruit juice obtained by mixing low-viscosity banana juice with passion fruits and pineapple juices, it was observed that the sensory analysis results showed a decrease in color and overall acceptance for all samples [48]. The poppy sherbet optimized by RSM showed a variation ranging from 0.04 to 0.26 points compared with the sensory analysis results obtained experimentally.

### 3.2. Color Properties in the Storage Process

The color parameters (L*, a*, b*, C, h) of optimized poppy sherbet samples on the 0th, 10th, 20th, and 30th days are provided in Table 6. It was observed that there was no statistically significant difference between the L*, a*, and h values of the 0th-day samples and the importance of the 30th-day samples (*p* > 0.05). No statistically significant difference was detected in the total color change (**ΔE)**. However, it was determined that the b* and C values significantly decreased compared with the 0th-day sample after a storage period of 30 days (*p* < 0.05). In a study by Ekici (2014) on poppy sherbet, it was observed that after 30 days of storage at 4 °C, the L* value decreased, while the C and h values increased compared with the 0th day [49]. This decrease in the L* value was observed to be similar our study. In a study by Lekjing and Venkatachalam (2021) determining various quality characteristics of wax apple cider vinegar, it was concluded that the b* value increased during the storage period, while the L* and a* values showed a tendency to decrease [50]. But our results regarding b* showed the opposite trend to that study. In a study conducted by Yıkmış et al. (2020) on sirkencübin syrup, it was observed that the L*, a*, and C values decreased in this syrup on the 30th day compared with the 0th day, while the b* and h values increased [46]. It was proposed that the Maillard reaction and the deterioration of anthocyanins were responsible for this drop in color values [51].

### 3.3. TPC and TFC in the Storage Process

In the medical and pharmaceutical fields, phenolic compounds have the potential to serve as bioactive agents to treat or prevent a variety of illnesses and improve human health [52]. The results of the TPC and TFC values in optimized poppy sherbet samples on the 0th, 10th, 20th, and 30th days are given in Table 7. When the total phenolic content (mg/L) of the optimized poppy sherbet samples on the 0th, 10th, 20th, and 30th days was examined, it was determined to be 47.36 ± 1.01 mg/L on the 0th day, 46.87 ± 0.48 mg/L on the 10th day, 43.76 ± 0.53 mg/L on the 20th day, and 39.66 ± 0.67 mg/L on the 30th day. It was observed that the total phenolic content significantly decreased in the 30th-day sample compared with the 0th-day sample (*p* < 0.05). Marsoul et al. (2020) reported that soxhlet and maceration *Papaver rhoeas* L. extracts were rich in total phenolic concentrations, with the values of 165.4 ± 3.84 mg GAE/g and 95.4 ± 2.42 mg GAE/g, respectively [17]; these values were quite high compared with our study. It may have been because dilution was achieved during the processing of the preparation of sorbet. Similarly, processing fruits into sorbets led to a loss of TPC (mandarin and orange (≈50%), strawberry (60–65%), mango and lemon (≈90%)) [44]. A study by Özen et al. (2020), on sour cherry vinegar, found that the total phenolic content increased on the 30th day compared with the 0th day; this increase was different to our study [53]. In a study by Shams Najafabadi et al. (2017), it was observed that the total phenolic content of concentrated jujube extract obtained by the conventional heating method and stored for 30 days at 4 °C decreased; this decrease was statistically significant (*p* < 0.05), which was parallel to our study [54]. In a study by Başyiğit and Hayoğlu (2019) on the production of microencapsulated licorice root sherbet using maltodextrin and Arabic gum via drying technology, it was found that there was no statistically significant difference between the 0th-day and 30th-day samples in terms of the total phenolic content of maltodextrin and control emulsions (*p* > 0.05) [55]. The general decrease in phenolic content was thought to be related to changes in the polymerization of phenolic compounds [56].

Among the phytoconstituents, flavonoids are known as natural antioxidants and found effective in the repair and prevention of oxidative damage [57]. TFC of the poppy sherbet sample (16.69 ± 0.40 mg CE/100 mL, 15.54 ± 0.37 mg CE/100 mL, 13.01 ± 0.30 mg CE/100 mL, and 12.28 ± 0.21 mg CE/100 mL) decreased significantly after the 10th day and continued to decrease until the end of the 30th day storage time. The Spanish sample of poppy had lower total phenolic and total flavonoid contents (25.86 ± 3.52 mg GAE/g extract and 12.00 ± 0.46 mg CE/g) than the Turkish poppy sherbet samples studied herein [58]. In the present study, although poppy sherbet was more dilute, there may have been differences due to soil fertility, different climatic conditions, and environmental and postharvest conditions [59]. Hmamou et al. (2023) showed that the TFC in leaf extract of poppy was 4.39 mg QE/g [15]. Krošlák et al. (2017) found that the TFC of fifteen poppy (*Papaver somniferum* L.) extracts (from Slovakia, Austria, Hungary, and the Czech Republic) was 1.17–11.28 mg QE/L [60]. In a study by Londoño et al. (2017) on the juice prepared using the pulp of a ripe mango variety, total flavonoid content was observed after a storage period of 30 days compared with the 0th day [61]. In the study conducted by Yıkmış and Tuğgüm (2019) on kombucha with black tea and varying proportions of purple basil tea, it was observed that the total flavonoid results of all kombucha samples decreased progressively over a 30-day storage period, similar our study [62].

### 3.4. Antioxidant Activity in the Storage Process

Because of the high concentrations of flavonoids and phenolics in poppy, it is thought to possess antioxidant capabilities [58]. The results of the DPPH and CUPRAC values in optimized poppy sherbet samples on the 0th, 10th, 20th, and 30th days are given in Table 7. Optimized poppy sherbet samples were found to have statistically higher DPPH and CUPRAC inhibition percentages in the 0th-day sample compared with the 30th-day sample (*p* < 0.05). Furthermore, in a recent Morocco study, the IC_50_ values for three poppy extracts (different geographical regions) were 0.27 ± 0.001 mg/mL, 0.30 ± 0.006, and 0.37 ± 0.003 [15]. In another study, the antioxidant activity of pure poppy-pollen extract in inhibiting DPPH 52.13 ± 2.34% was found [63]. Ekici et al. (2018) studied engagement sorbet that made dry black grapes, corn poppy flowers, sugar, and water. They found antioxidant activity was 31.19 mg AAE/L in engagement sorbet [45]. In a study by Kang et al. (2020) on five commercial vinegars, the decrease in DPPH inhibition percentage after a 12-month storage period in the fourth and fifth brand commercial vinegars was similar to our study [64]. In an opposite study, Machado et al. (2019) found that the antioxidant activity, determined by the DPPH method, in probiotic juçara and banana sorbet during −20 °C storage showed a significant increase [65]. Also, Evrendilek et al. (2023) detected that the antioxidant capacity of the pulsed electric field-treated (6.90 kV/cm, 756.00 µs, and 7.48 °C) licorice root sherbet samples at 4 °C did not significantly change within the 40th-day storage [66]. In a study by Güler (2021) to determine the usability of sour grape concentrate in beverages, it was observed that the DPPH inhibition percentage decreased in the beverage with added sour grape concentrate after a 6-month storage period at 4 °C [67]. It is believed that the decrease in antioxidant activity may be associated with the degradation of anthocyanins [68].

### 3.5. Total Anthocyanin Content

Anthocyanin provides red, blue, and purple colors to plants in a water-soluble pigment [69]. The red color of poppy is related to the inclusion of various anthocyanins such as coumarin, rutin, cyanidol, malvidin, luteolinidin, and vitexin [70]. Velickovic et al. (2019) reported that the most represented anthocyanins in the extracts of Papaver rhoeas L. are cyanidin-3-O-rutinoside, cyanidin-3-O-glucoside, delphinidin-3-p-coumaroylglucoside, delphinidin-3-O-glucoside, peonidin-3-O-glucoside, petunidin-3-acetylglucoside, and petunidin-3-O-glucoside [71]. The total anthocyanin content of optimized poppy sherbet samples is given in Table 7. Optimized poppy sherbet samples were found to have decreased total monomeric anthocyanin content (mg Cy-3-gly/100 mL) during the 30-day storage period, with values of 127.23 ± 1.57, 116.87 ± 2.20, 98.22 ± 1.14, and 92.45 ± 1.08 mg Cy-3-gly/100 mL for the 0th, 10th, 20th, and 30th-day samples, respectively. Kostic et al. (2010) reported lower total anthocyanin content of 5.193 ± 0.082 from *Papaver rhoeas* L. ethanol extracts compared with the present study, which may have been reasoned by heating and drying processes [72]. In a study by Ergün et al. (2017) on sherbet prepared from black mulberry and blueberry fruits, it was found that the anthocyanin content of black mulberry and blueberry sherbet decreased on the 30th day compared with the 0th day; this decrease was statistically significant (*p* < 0.05), similarly to our study [73]. In a study by Ertan et al. (2018) on strawberry and sour cherry nectars prepared by adding honey, sucrose, and maltose syrup, a decrease in anthocyanin content was observed in all samples during 168 days [74]. In a study by Mgaya-Kilima et al. (2015) on a mixture of roselle extract and mango juice stored at 4 °C and 28 °C, a decrease in total monomeric anthocyanin content was observed after a 6-month storage period at 4 °C [75].

### 3.6. Analysis of Phenolic Compounds

Alkaloids are the most abundant metabolites in the *Papaveraceae* family. Alkaloids have antioxidant and antimicrobial activities [15]. The polyphenol results of poppy sherbet samples are shown in Table 8. Optimized poppy sherbet samples were analyzed for the presence of 17 phenolic compounds. Gallic acid, protocatechuic acid, gentisic acid, and trans-cinnamic acid compounds were detected in optimized poppy sherbet 0th, 10th, 20th, and 30th-day samples. Among these compounds, gallic acid was found in the highest amount. Although there was no significant difference (*p* > 0.05), a slight decrease in the amount of gallic acid was observed in the 30th-day sample (23.403 ± 0.343 μg/mL) compared with the 0th-day sample (23.886 ± 0.164 μg/mL). However, the total phenolic content decreased during the storage period. In a study by Kafadar et al. (2021) on some traditional Turkish sherbets, it was found that the levels of gallic acid and protocatechuic acid increased during a 90-day storage period at 4 °C for engagement sherbet, while the level of coumarin remained unchanged [76]. In another study, it was found that the levels of gallic acid, protocatechuic acid, p-coumaric acid, and coumarin increased after a 90-day storage period at 4 °C for traditional tamarind sherbet [77]. These two investigations contradict our findings because they found that gallic acid and protocatechuic acid levels rose after a particular amount of storage time. However, compared with the 0th-day sample, our research revealed a modest decrease in these chemicals in the 30-day sample.

**Figure 5:**
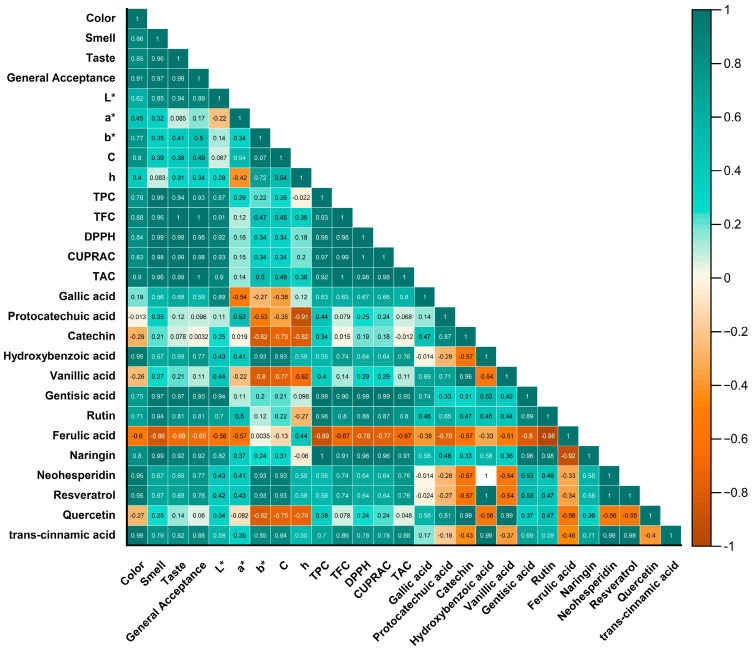
Pearson’s correlation coefficients of TPC, TFC, TAC, DPPH, CUPRAC, sensory, color, and phenolic compounds.

The correlation analysis was used to measure the linear association between variables. Pearson’s positive correlation coefficients (R^2^) among the TPC were significantly correlated with naringin (1), rutin (0.96), and gentisic acid (0.98) (Figure 5). TFC was extremely positively correlated with smell (0.96), gentisic acid (0.96), and naringin (0.91). Finally, positive correlations among phenolic compounds and sensory attributes were found in some cases, e.g., gentisic acid and smell–taste (r = 0.97), gentisic acid and general acceptance (r = 0.95), rutin and smell (r = 0.94), naringin and smell (r = 0.99), and naringin and taste–general acceptance (0.92). The other significant correlations were color–resveratrol (0.95), color–neohesperidin (0.95), color–hydroxybenzoic acid (0.95), and color–total monomeric anthocyanin (0.90).

## 4. Conclusions

Edible plants that are non-toxic and have various health benefits are consumed in different regions of the world as part of human nutrition. The edible flower known as the poppy (*Papaver rhoeas*) is found growing all over the world. According to our knowledge, the current work is the first to use RSM modeling tools to optimize the constituent amounts for good-tasting poppy sherbet (dry poppy flower, sugar, and citric acid). The ratio of the three elements for the best sensory value was determined using RSM. This optimum poppy sherbet was produced with 0.26 g of dried poppy flower, 4.29 g of sucrose, and 0.15 g of citric acid. Optimized poppy sherbet was determined by RSM to have sensory analysis parameters (8.55 for color; 7.19 for smell; 8.38 for taste; 7.98 for general acceptance). Gallic acid was found in the highest amount. There was no significant difference between the 0th day (23.886 ± 0.164 μg/mL, 1.146 ± 0.048 μg/mL) and 30th day (23.403 ± 0.343 μg/mL, 1.047 ± 0.038 μg/mL) sample in gallic acid and protocatechuic acid. Pearson’s positive correlations among phenolic compounds and sensory attributes were found in some cases. TFC, TPC, CUPRAC, DPPH, TAC, and color values were found to decrease as the storage period increased (*p* < 0.05). In conclusion, these findings suggest that hops from the edible plant poppy may be a potent bioactive source, although some show a decrease after 30 days of storage. Based on the results obtained in this study, more studies with more panelists are needed to better understand the sensory properties relationships of bioactive compounds. In addition, food safety factors such as pesticide content and heavy metal contamination in poppy flowers need to be defined in detail. It is concluded that the results of this study should lead to future in vivo studies.

## Figures and Tables

**Table 1:** Levels of independent variables in the RSM.

Independent Variables		Levels		
	Codes	−1	0	1
Dried poppy flower (g/100 mL)	X_1_	0.1	0.2	0.3
Sucrose (g/100 mL)	X_2_	3	4	5
Citric acid (g/100 mL)	X_3_	0.05	0.10	0.15

**Table 2:** Measured responses used in the experimental design for RSM.

Sample	Encoded Independent Variables			Dependent Variables							
				Response 1		Response 2		Response 3		Response 4	
				Color		Smell		Taste		General Acceptance	
	X_1_	X_2_	X_3_	Experimental Data	RSM Predicted	Experimental Data	RSM Predicted	Experimental Data	RSM Predicted	Experimental Data	RSM Predicted
1	0.10	3.00	0.10	6.56	6.58	5.48	5.46	6.42	6.43	6.10	6.07
2	0.30	3.00	0.10	8.08	8.18	6.75	6.78	7.92	7.97	7.52	7.52
3	0.10	5.00	0.10	7.40	7.30	6.20	6.18	7.26	7.20	6.89	6.89
4	0.30	5.00	0.10	8.33	8.30	6.95	6.96	8.15	8.13	7.74	7.77
5	0.10	4.00	0.05	6.87	6.84	5.76	5.74	6.73	6.68	6.42	6.41
6	0.30	4.00	0.05	8.34	8.23	6.81	6.75	7.97	7.87	7.57	7.53
7	0.10	4.00	0.15	7.19	7.29	5.97	6.03	6.97	7.07	6.64	6.68
8	0.30	4.00	0.15	8.47	8.50	7.10	7.12	8.30	8.35	7.89	7.90
9	0.20	3.00	0.05	7.60	7.60	6.18	6.21	7.23	7.27	6.86	6.91
10	0.20	5.00	0.05	7.97	8.09	6.77	6.81	7.80	7.91	7.52	7.53
11	0.20	3.00	0.15	8.16	8.03	6.74	6.69	8.00	7.88	7.33	7.32
12	0.20	5.00	0.15	8.39	8.38	7.03	6.99	8.22	8.17	7.81	7.76
13	0.20	4.00	0.10	8.16	8.16	6.99	6.98	8.00	8.00	7.77	7.77
14	0.20	4.00	0.10	8.11	8.16	6.92	6.98	7.92	8.00	7.74	7.77
15	0.20	4.00	0.10	8.23	8.16	7.02	6.98	8.08	8.00	7.80	7.77

X_1_—dried poppy flower; X_2_—sucrose; X_3_—citric acid.

**Table 3:** Analysis of variance in a regression model of the central combination test.

Source	DF	Adj SS	Adj MS	F-Value	*p*-Value	Adj SS	Adj MS	F-Value	*p*-Value	Adj SS	Adj MS	F-Value	*p*-Value	Adj SS	Adj MS	F-Value	*p*-Value
		Color				Smell				Taste				General Acceptance			
Model	9	4.83	0.54	30.88	0.001	3.76	0.42	92.02	0.000	4.76	0.53	35.13	0.001	4.63	0.51	221.58	0.000
Linear	3	3.98	1.33	76.19	0.000	2.82	0.94	207.08	0.000	3.89	1.30	86.07	0.000	3.51	1.17	503.92	0.000
X_1_	1	3.37	3.37	193.52	0.000	2.20	2.20	484.41	0.000	3.07	3.07	203.71	0.000	2.72	2.72	1173.20	0.000
X_2_	1	0.35	0.35	20.36	0.006	0.41	0.41	89.26	0.000	0.43	0.43	28.86	0.003	0.58	0.58	249.23	0.000
X_3_	1	0.26	0.26	14.71	0.012	0.22	0.22	47.56	0.001	0.39	0.39	25.65	0.004	0.21	0.21	89.32	0.000
Square	3	0.76	0.25	14.53	0.007	0.85	0.28	62.15	0.000	0.75	0.25	16.56	0.005	1.03	0.34	147.58	0.000
X_1_ × X_1_	1	0.72	0.72	41.35	0.001	0.75	0.75	164.44	0.000	0.72	0.72	47.54	0.001	0.85	0.85	364.53	0.000
X_2_ × X_2_	1	0.06	0.06	3.62	0.116	0.12	0.12	26.82	0.004	0.06	0.06	3.73	0.111	0.19	0.19	82.24	0.000
X_3_ × X_3_	1	0.00	0.00	0.01	0.927	0.05	0.05	11.24	0.020	0.02	0.02	1.05	0.352	0.10	0.10	41.89	0.001
2-Way Interaction	3	0.10	0.03	1.92	0.244	0.09	0.03	6.84	0.032	0.12	0.04	2.76	0.151	0.09	0.03	13.25	0.008
X_1_ × X_2_	1	0.09	0.09	4.99	0.076	0.07	0.07	15.14	0.012	0.09	0.09	6.04	0.057	0.08	0.08	35.39	0.002
X_1_ × X_3_	1	0.01	0.01	0.50	0.509	0.00	0.00	0.29	0.615	0.00	0.00	0.15	0.716	0.00	0.00	0.94	0.376
X_2_ × X_3_	1	0.00	0.00	0.28	0.618	0.02	0.02	5.10	0.073	0.03	0.03	2.09	0.208	0.01	0.01	3.43	0.123
Error	5	0.09	0.02			0.02	0.00			0.08	0.02			0.01	0.00		
Lack-of-Fit	3	0.08	0.03	6.86	0.130	0.02	0.01	2.16	0.332	0.06	0.02	3.26	0.244	0.01	0.00	3.63	0.223
Pure Error	2	0.01	0.00			0.01	0.00			0.01	0.01			0.00	0.00		
Total	14	4.92				3.79				4.84				4.64			
R^2^		98.23%				99.40%				98.44%				99.75%			
Adj. R^2^		95.05%				98.32%				95.64%				99.30%			
Pred. R^2^		73.88%				92.34%				78.73%				96.53%			

X_1_—dried poppy flower; X_2_—sucrose; X_3_—citric acid; df—degrees of freedom; R^2^—coefficient of determination. *p* < 0.05, significant differences; *p* < 0.01, very significant differences.

**Table 4:** Maximum optimization values according to RSM.

Variable	Setting			
X_1_—Dried poppy flower (gram)	0.26			
X_2_—Sucrose (gram)	4.29			
X_3_—Citric acid (gram)	0.15			
Response	Fit	SE Fit	%95 CI	%95 PI
General acceptance	7.98	0.03	(7.8966; 8.0653)	(7.8311; 8.1308)
Taste	8.38	0.08	(8.1673; 8.5973)	(8.0005; 8.7641)
Smell	7.19	0.04	(7.0757; 7.3118)	(6.9841; 7.4034)
Color	8.55	0.08	(8.3216; 8.7836)	(8.1424; 8.9628)

CI—confidence interval; PI—prediction interval.

**Table 5:** Sensory properties of optimized poppy sherbet during storage.

Sensory Features	Storage Time	Sensory Analysis Results
Color	0 day	8.44 ± 0.64 ^a^
	10 day	8.02 ± 0.87 ^b^
	20 day	7.96 ± 0.60 ^b^
	30 day	7.82 ± 0.75 ^b^
Smell	0 day	7.14 ± 0.57 ^a^
	10 day	7.06 ± 0.55 ^ab^
	20 day	6.92 ± 0.70 ^ab^
	30 day	6.74 ± 0.75 ^b^
Taste	0 day	8.12 ± 1.10 ^a^
	10 day	8.02 ± 0.84 ^ab^
	20 day	7.68 ± 1.00 ^ab^
	30 day	7.58 ± 0.81 ^b^
General Acceptance	0 day	8.02 ± 0.98 ^a^
	10 day	7.90 ± 0.54 ^a^
	20 day	7.70 ± 0.79 ^a^
	30 day	7.62 ± 1.01 ^a^

The results are presented as mean ± standard deviation. Different letters in the rows indicate significant differences (*p* < 0.05).

**Table 6:** Color attributes of optimized poppy sherbet during storage.

Color Properties	Storage Time			
	0 Day	10 Day	20 Day	30 Day
L*	41.93 ± 0.69 ^ab^	42.37 ± 1.20 ^a^	40.41 ± 0.22 ^b^	40.36 ± 0.26 ^b^
a*	7.06 ± 0.23 ^a^	6.75 ± 0.26 ^a^	7.16 ± 0.12 ^a^	6.75 ± 0.13 ^a^
b*	12.21 ± 0.05 ^a^	11.12 ± 0.20 ^c^	11.28 ± 0.04 ^bc^	11.54 ± 0.03 ^b^
C	14.10 ± 0.13 ^a^	13.01 ± 0.31 ^b^	13.36 ± 0.06 ^b^	13.37 ± 0.09 ^b^
h	59.95 ± 0.81 ^a^	58.76 ± 0.53 ^ab^	57.60 ± 0.48 ^b^	59.71 ± 0.42 ^a^
ΔE		1.91 ± 0.74 ^a^	1.87 ± 0.70 ^a^	1.78 ± 0.80 ^a^

L*—luminosity and lightness; a*—greenness and redness; b*: ΔE: color change blueness and yellowness C (chroma) h (hue angle). The results are presented as mean ± standard deviation. Different letters in the rows indicate significant differences (*p* < 0.05).

**Table 7:** TPC, TFC, DPPH, CUPRAC, and TAC analysis results of optimized poppy sherbet during storage.

Analyses	Storage Time			
	0 Day	10 Day	20 Day	30 Day
TPC (mg GAE/100 mL)	47.36 ± 1.01 ^a^	46.87 ± 0.48 ^a^	43.76 ± 0.53 ^b^	39.66 ± 0.67 ^c^
TFC (mg CE/100 mL)	16.69 ± 0.40 ^a^	15.54 ± 0.37 ^b^	13.01 ± 0.30 ^c^	12.28 ± 0.21 ^c^
DPPH (% inhibition)	56.06 ± 0.45 ^a^	55.66 ± 0.24 ^a^	54.03 ± 0.15 ^b^	53.05 ± 0.34 ^c^
CUPRAC (% inhibition)	58.84 ± 0.46 ^a^	58.44 ± 0.31 ^a^	56.73 ± 0.16 ^b^	55.78 ± 0.17 ^c^
TAC (mg Cy-3-gly/100 mL)	127.23 ± 1.57 ^a^	116.87 ± 2.20 ^b^	98.22 ± 1.14 ^c^	92.45 ± 1.08 ^d^

The results are presented as mean ± standard deviation. Different letters in the rows indicate significant differences (*p* < 0.05). TPC (total phenolic content), TFC (total flavonoid content), DPPH (2,2-diphenyl-1-picrylhydrazyl), CUPRAC (cupric reducing antioxidant capacity), and TAC (total monomeric anthocyanin content).

**Table 8:** Results of phenolic compounds of optimized poppy sherbet during storage.

Phenolic Compounds (μg/mL)	Storage Time			
	0 Day	10 Day	20 Day	30 Day
Gallic acid	23.886 ± 0.164 ^a^	25.137 ± 0.222 ^b^	23.191 ± 0.126 ^a^	23.403 ± 0.343 ^a^
Protocatechuic acid	1.146 ± 0.048 ^ab^	1.264 ± 0.017 ^bc^	1.350 ± 0.021 ^c^	1.047 ± 0.038 ^a^
Catechin	ND	0.289 ± 0.003 ^a^	0.234 ± 0 ^b^	ND
Hydroxybenzoic acid	0.024 ± 0.002 ^a^	ND	ND	ND
Vanillic acid	ND	0.041 ± 0.008 ^a^	0.021 ± 0.002 ^b^	0.001 ± 0 ^c^
Gentisic acid	0.237 ± 0.016 ^a^	0.240 ± 0.008 ^a^	0.161 ± 0.004 ^b^	0.114 ± 0.001 ^c^
p-coumaric acid	ND	ND	ND	ND
Rutin	1.350 ± 0.038 ^a^	1.255 ± 0.014 ^b^	1.073 ± 0.014 ^c^	ND
Ferulic acid	ND	ND	ND	0.217 ± 0.008 ^a^
Naringin	0.267 ± 0.008 ^a^	0.234 ± 0.006 ^b^	0.155 ± 0.004 ^c^	ND
o-coumaric acid	ND	ND	ND	ND
Neohesperidin	0.844 ± 0.012 ^a^	ND	ND	ND
Coumarin	ND	ND	ND	ND
Resveratrol	0.053 ± 0.002 ^a^	ND	0.001 ± 0 ^b^	ND
Quercetin	ND	1.237 ± 0.017 ^a^	0.828 ± 0.006 ^b^	ND
trans-cinnamic acid	3.214 ± 0.013 ^a^	0.758 ± 0.007 ^b^	0.252 ± 0.016 ^c^	0.079 ± 0.006 ^d^
Total	31.388 ± 0.413 ^a^	30.399 ± 0.380 ^a^	27.152 ± 0.016 ^b^	24.945 ± 0.247 ^c^

The results are presented as mean ± standard deviation. Different letters indicate significant differences among the values within the same row (*p* < 0.05). ND—not detected.

## Data Availability

The datasets generated for this study are available on request to the corresponding author.
